# SENP2 regulates UCP1-dependent thermogenesis in brown adipocytes via deSUMOylation of ERRα

**DOI:** 10.1038/s12276-025-01458-5

**Published:** 2025-06-27

**Authors:** Hun Jee Choe, Ji Seon Lee, Jong Yoen Park, Seung-Ah Lee, Young Joo Park, Sung Soo Chung, Kyong Soo Park

**Affiliations:** 1 Department of Internal Medicine, Seoul National University College of Medicine, Seoul, Republic of Korea; 2 Department of Internal Medicine, Hallym University Dongtan Sacred Heart Hospital, Hallym University College of Medicine, Hwaseong, Republic of Korea; 3 Biomedical Research Institute, Seoul National University Hospital, Seoul, Republic of Korea; 4 Department of Internal Medicine, Konkuk University Medical Center, Seoul, Republic of Korea; 5 Genomic Medicine Institute, Seoul National University Medical Research Center, Seoul, Republic of Korea; 6 Division of Endocrinology and Metabolism, Department of Internal Medicine, Seoul National University Hospital, Seoul, Republic of Korea

**Keywords:** Sumoylation, Metabolic syndrome

## Abstract

Brown adipose tissue (BAT) is responsible for energy homeostasis and adaptive thermogenesis. SUMO-specific protease 2 (SENP2) plays an essential role in adipogenesis; however, the role of SENP2 in BAT metabolism has not been explored. Here we investigated the role of SENP2 in mature brown adipocytes with a brown adipocyte-specific SENP2 knockout (*Senp2*-BKO) mouse model generated using the uncoupling protein 1 (*Ucp1*)-Cre. High-fat diet-induced insulin resistance was aggravated in *Senp2*-BKO mice compared with control mice. In *Senp2*-BKO mice, adaptive thermogenesis upon acute cold exposure was impaired and UCP1 expression was barely induced upon cold or β-adrenergic stimulation. SENP2-mediated deSUMOylation of estrogen-related receptor alpha (ERRα) significantly enhanced *Ucp1* promoter activity through activation of the ERRα/PGC1α complex. The absence of SENP2 inhibited formation of ERRα, cAMP-response element-binding protein (CREB) and RNA Polymerase II transcriptional complex at the *Ucp1* promoter following β3-adrenergic stimulation. In addition, SUMOylation of ERRα severely interfered with binding of ERRα to its DNA-binding site (ERRE) in the promoter of *Ucp1*. Our findings revealed that SENP2 plays a role in the metabolic flexibility and thermogenic efficiency of BAT, particularly in response to β3-adrenergic activation.

## SENP2 regulates brown fat thermogenesis and metabolic flexibility

Brown adipose tissue helps burn energy and generate heat, unlike white fat, which stores energy. This study explores how the SENP2 protein affects the ability of brown adipose tissue to generate heat and manage energy. The researchers used mice genetically modified to lack SENP2 specifically in their brown fat cells. They found that these mice had trouble maintaining body temperature in the cold and showed signs of insulin resistance when fed a high-fat diet. The study involved exposing these mice to cold temperatures and analyzing their brown fat tissue. The researchers discovered that SENP2, an enzyme regulating a process called deSUMOylation, influences the activity of ERRα, a protein crucial for activating genes involved in heat production. This summary was initially drafted using artificial intelligence, then revised and fact-checked by the author.

## Introduction

Brown adipose tissue (BAT) is thermogenic fat that dissipates energy as heat, whereas white adipose tissue (WAT) stores energy. Activated BAT contributes to non-shivering thermogenesis and increases energy expenditure by utilizing free fatty acids and glucose^1,2^. As substantial evidence indicates that metabolically active BAT is present in adult humans, BAT may be a therapeutic target for metabolic disease^3–6^. Cold-induced thermogenesis in BAT is mediated through the β3-adrenergic signaling pathway, in which mitochondrial uncoupling protein 1 (UCP1) plays a central role. Upon cold exposure, norepinephrine binds to the β3-adrenergic receptor to increase the level of cAMP, which activates protein kinase A (PKA). PKA activates cAMP-response element-binding protein (CREB), which translocates into the nucleus to enhance *Ucp1* transcription. PKA also activates p38 and hormone-sensitive lipase, which further enhances thermogenesis by increasing thermogenic gene expression and providing energy substrates^7^.

Post-translational modification of proteins by the small ubiquitin-related modifier (SUMO) modulates the function, localization and stability of target proteins^8^, thereby regulating various cellular processes^9–11^. SUMO modification can be reversed by SUMO-specific proteases (SENPs) that remove SUMO from target proteins. Previously, we showed that SUMO-specific protease 2 (SENP2) plays important roles in various metabolic contexts. In skeletal muscle, SENP2 increases expression of fatty acid oxidation-associated enzymes, such as carnitine palmitoyl transferase-1 and long-chain acyl-CoA synthetase 1, by deSUMOylating peroxisome proliferator-activated receptor (PPAR)-δ and PPAR-γ. In transgenic mice overexpressing muscle-specific SENP2, high-fat diet (HFD)-induced obesity was ameliorated^12^. In pancreatic β cells, SENP2 improves mitochondrial function and insulin secretion upon metabolic stress by deSUMOylating dynamin-related protein 1^13^.

Although SENP2 is highly expressed in adipose tissue^14^, our current understanding of the role of SENP2 in adipose tissue metabolism, particularly in BAT, remains incomplete. SENP2 expression and its role in early adipogenesis have been documented^15,16^; however, the role of SENP2 in mature brown adipocytes under various metabolic stressors has not yet been fully explored. In WAT, SENP2 is intimately involved in adipogenesis and maintaining white adipocyte identity by stabilizing CCAAT/enhancer binding protein β^14,15^. Specifically, *Senp2* knockout in both BAT and WAT using *Adipoq*-Cre (*Senp2*-aKO) induces browning in WAT, which exerts a beneficial effect on whole-body metabolism. Intriguingly, while the metabolic phenotype improves in the *Senp2*-aKO due to the browning of WAT, whitening of BAT was observed upon HFD feeding^17^. These data suggest that SENP2 plays an essential role in the metabolic adaptability of BAT, particularly in lipid utilization during energy overload.

In this study, using brown adipocyte-specific SENP2 knockout (*Senp2*-BKO) mice, we demonstrated that SENP2 controlled BAT thermogenesis during acute cold stimulation and positively regulated energy balance during energy overload. We also showed that SENP2-mediated deSUMOylation of estrogen-related receptor alpha (ERRα) enhanced *Ucp1* expression upon exposure to cold stimuli. Collectively, our data suggest that SENP2 plays an important role in maintaining the healthy metabolic phenotype of BAT.

## Materials and methods

### Generation of brown adipocyte-specific *Senp2* knockout mice

*Senp2*^*flox/+*^ mice were generated in the inGenious Targeting Laboratory, as described previously^18^. *Senp2* brown adipocyte-specific knockout (*Senp2*-BKO) mice were generated by sequentially mating *Senp2*^*flox/flox*^ with *Ucp1*-Cre transgenic mice (Jackson lab).

### Mice and metabolic analysis

All aspects of animal care and experiments were conducted in accordance with the Guide for the Care and Use of Laboratory Animals of the National Institutes of Health and approved by the Institutional Animal Care and Use Committee of Seoul National University Bundang Hospital, Korea (permit no. BA-2107-324-070). All animals were housed at 22–24 °C, with a 12:12 h light–dark cycle and *ad libitum* access to standard pelleted chow or HFD (58 kcal% fat with sucrose, D12331; Research Diets) and water. Eight-week-old mice were fed a HFD for 12 weeks. Body weight was measured weekly, and body composition was measured by body composition analyzer (Minispec LF50, Bruker). For the glucose tolerance test (GTT), glucose (1 g/kg body weight) was administered via intraperitoneal injection (i.p.) after a 16 h fasting period. For the insulin tolerance test (ITT), mice that had fasted for 6 h received an injection of human insulin (1 U/kg body weight). Serum glucose levels were measured using an OneTouch Ultra glucometer (LifeScan). For the acute cold exposure experiment, mice were kept at room temperature or at 4 °C, as specified, and body temperatures were recorded rectally every hour for 5 h using a digital thermometer. To simulate cold exposure, 10 mg/kg of CL316,243 (Sigma-Aldrich) was administered, and the mice were euthanized for BAT dissection and RNA extraction 6 h post injection. Metabolic cage experiments were conducted using a Comprehensive Lab Animal Monitoring System (Columbus Instruments, Columbus).

### Histological analysis

Tissues were fixed in 4% formaldehyde, embedded in paraffin and sectioned. Sections were subjected to hematoxylin and eosin (H&E) staining. Immunohistochemistry was performed with antibodies against UCP1 and Perilipin 1 (Abcam).

### mRNA sequencing and data analysis

Total RNA was obtained from BAT of control or *Senp2*-BKO mice. RNA-sequencing libraries were prepared by TruSeq Stranded mRNA LT Sample Prep kit (Illumina Inc.). The libraries were sequenced, and the reads were aligned to mouse transcriptome (UCSC gene) and genome (mm10) references, respectively, using HISAT2 version 2.1.0 and Bowtie2 2.3.4.1. Trimming tasks for Illumina paired-end and single-ended data for each sample’s FASTQ files were performed using Trimmomatic 0.38. Transcript assembly was performed using the StringTie program and relative log expression normalization was processed after filtering the genes with low quality. Specifically, genes with more than 50% of 0 read counts were excluded from the analysis. From the read counts and transcripts per kilobase million, differential gene expression analysis was performed with DESeq2 R statistical package using the criteria of |log2 fold change | ≥2 and nbinomWaldTest raw *P* < 0.05. Gene set enrichment analysis was utilized to retrieve Gene Ontology and Kyoto Encyclopedia of Genes and Genomes (KEGG) pathway data.

### Real-time qPCR

Total RNA was isolated using TRIzol (Invitrogen) according to the manufacturer’s instructions. Real-time qPCR was performed (in duplicates) using the SYBR master mix (Takara) and the ABI 7500 Real-time PCR system (Applied Biosystem). Each cycle threshold (Ct) value was subtracted from Ct value of GAPDH or β-actin (ΔCt), and then subtracted from the value of each control set (ΔΔCt). Relative mRNA levels were expressed as 2^−ΔΔCt^. Sequences of the primers used for qPCR are presented in the Supplementary Table 1.

### Cell culture

The mouse brown pre-adipocyte cell line, derived from C57BL/6J mice and immortalized using an SV40 viral vector with puromycin selection^19^, was a generous gift from Dr. Shingo Kajimura. This cell line was cultured in high glucose Dulbecco’s modified Eagle medium (DMEM) (Hyclone), supplemented with 10% fetal bovine serum (FBS) and 100 U/ml penicillin–streptomycin (Thermo Fisher Scientific) at 37 °C in a humidified atmosphere containing 5% CO_2_. To induce differentiation, 95% confluent cells from this established cell line were treated with a differentiation medium composed of DMEM containing 10% FBS, 1% penicillin–streptomycin, 0.5 μM rosiglitazone (Sigma-Aldrich), 1 nM T3 (triiodothyronine) (Sigma-Aldrich), 850 nM insulin, 125 nM indomethacin, 2 μg/ml dexamethasone and 0.5 mM isobutylmethylxanthine. Two days post-induction, the medium was replaced with maintenance medium, which included 10% FBS, 1% penicillin–streptomycin, 1 nM T3 and 850 nM insulin. The cells from the mouse brown adipocyte cell line reached full differentiation 6 days after the initiation of the differentiation process.

### Transfection of siRNAs

Small interfering RNAs (siRNAs) of SENP2, ERRα, PPARα, PPARγ and PGC1α were purchased from Dharmacon. Nonspecific siRNAs (siNS) were purchased from BIONEER. To enhance transfection efficiency without interfering with adipogenesis, target genes were knocked down on day 4 of brown pre-adipocyte differentiation using the reverse siRNA transfection method. Lipofectamine RNAiMAX (Invitrogen) and siRNAs were diluted separately in serum-free DMEM, and 250 μl of each reagent was added to gelatin-coated 12-well cell culture plates to be incubated for 25 min at room temperature. The final concentrations of lipofectamine RNAiMAX and siRNA were 2.5 μl/ml and 50 nM, respectively. During incubation, cells were detached with 0.05% Trypsin–EDTA (25300054, Gibco) for 2 min, centrifuged (3,000 rpm, 5 min) and resuspended in the culture medium containing 2 nM T3 and 1,700 nM insulin. On the top of the pre-incubated siRNA–RNAiMAX complex, 500 μl of the cell suspension were added. The final concentrations of T3 and insulin were the same as the maintenance media. The replated cells were further cultured for 2 days without changing media and were collected for analysis at day 6 of differentiation.

### Transient transfection and luciferase reporter assay

A DNA fragment encompassing the *Ucp1* promoter region (ranging from −2,803 bp to +213 bp relative to the transcription start site) was cloned into the pGL2-basic vector to generate the m*Ucp1*-Luc reporter vector. The distal ERRE mutant m*Ucp1*-Luc reporter vector (m*Ucp1* mt1-Luc) was created by modifying the sequence at –2,407 bp/–2,390 bp ‘GCA GCA AGG TCA ACC CTT’ to ‘GCA GCA TCC ACA ACC CTT’. Similarly, the proximal ERRE mutant m*Ucp1*-Luc reporter vectors (m*Ucp1* mt2-Luc) were developed through site-directed mutagenesis, altering the sequence at –687 bp/–670 bp ‘CCC TCT GTC CTT CCA GGG’ to ‘CCC TCT GAG GTT CCA GGG’. Mutant forms of HA-PGC1α (PGC1α K183R) and HA-ERRα (ERRα K14R) were produced using the Quick Change Site-directed Mutagenesis kit (Agilent Technologies), as described previously^17^.

For transfection, Cos-7 cells or brown adipocytes were incubated with 0.3 μg of the *Ucp1* promoter-Luc vector, 0.1 μg of expression vectors for PGC1α, ERRα, PPARγ, RXRα and SENP2, and 0.1 μg of pRSV-β-galactosidase as indicated, using Lipofectamine Plus (Thermo Fisher Scientific). To stimulate the cells, 10 μM forskolin (Sigma-Aldrich) was added for 18 h before collecting. Cells were collected 1 day post-transfection using Reporter Lysis Buffer (Promega Corporation), and luciferase activity was measured with the Luciferase Assay System (Promega Corporation) using a Lumat LB9507 luminometer (Berthold Technologies). Luciferase activity was normalized to β-galactosidase activity for accurate quantification.

### Western blot analysis

For western blot analysis of tissue and cell lysates, samples were prepared in RIPA buffer (Merck Millipore) supplemented with protease inhibitors. Proteins were separated by SDS–PAGE and transferred onto nitrocellulose membranes (Whatman). After incubation with specific antibodies, bands were visualized by Enhanced Chemiluminescence (Pierce) and Amersham Imager 680 Blot and Gel imagers (GE Healthcare Life Sciences). Antibodies against PGC1α (Santa Cruz Biotechnology Inc.), ERRα (Abcam), UCP1 (Abcam) and γ-tubulin (Sigma-Aldrich) were used for western blotting.

### ChIP-linked qPCR

Chromatin immunoprecipitation (ChIP) assays were performed using the EZ-ChIP kit (Merck Millipore) according to the manufacturer’s instructions. Control or *Senp2*-BKO mice were administered with 10 mg/kg of CL316,243, and BAT was obtained 6 h after injection. BAT of control or *Senp2*-BKO mice were crosslinked with 1% formaldehyde for 15 min and lysed with the lysis buffer. Nuclear extracts were sonicated on ice to generate DNA fragments with an average length of 200–500 bp. Ten percent of each sample was saved as input fraction. Nuclear extracts were immunoprecipitated with antibodies against ERRα (Abcam), CREB (Cell Signaling Technology), Pol II (Santa Cruz Biotechnology Inc.) or control IgG (Santa Cruz Biotechnology Inc.). Real-time qPCR was performed using the primers for the ERRE site of the *Ucp1* promoter.

### EMSAs

Electrophoretic-mobility shift assays (EMSAs) were performed using LightShift Chemiluminescent Electrophoretic-Mobility Shift Assay kit (Thermo Fisher Scientific). A probe, containing the sequence of ERRE in the *Ucp1* enhancer region, was labeled with biotin and incubated with nuclear extracts from Cos-7 transfected with ERRα or ERRα-SUMO expression vectors in the binding buffer. The probe–protein complexes were separated using a 6% polyacrylamide gel and transferred onto nylon membranes. The membrane was detected by Chemiluminescent Nucleic Acid Detection Module (Thermo Fisher Scientific). The probe sequences are listed in Supplementary Table 1.

### Statistical analysis

All statistical analyses were performed using IBM SPSS Statistics version 27.0 (IBM Corp.) and GraphPad Prism version 7.0 for Windows (GraphPad Software, www.graphpad.com). Group differences were assessed using Student’s *t*-test or analysis of variance (ANOVA) for continuous variables and the chi-squared test for categorical variables. For multiple comparison adjustments, Tukey’s honestly significant difference (HSD) correction was applied during post hoc analyses after calculating *P* values through ANOVA. Two-sided *P* values <0.05 were considered statistically significant.

## Results

### Brown fat-specific loss of SENP2 does not affect adipogenesis but affects expression of genes involved in thermogenesis and oxidative phosphorylation

**Fig. 1:**
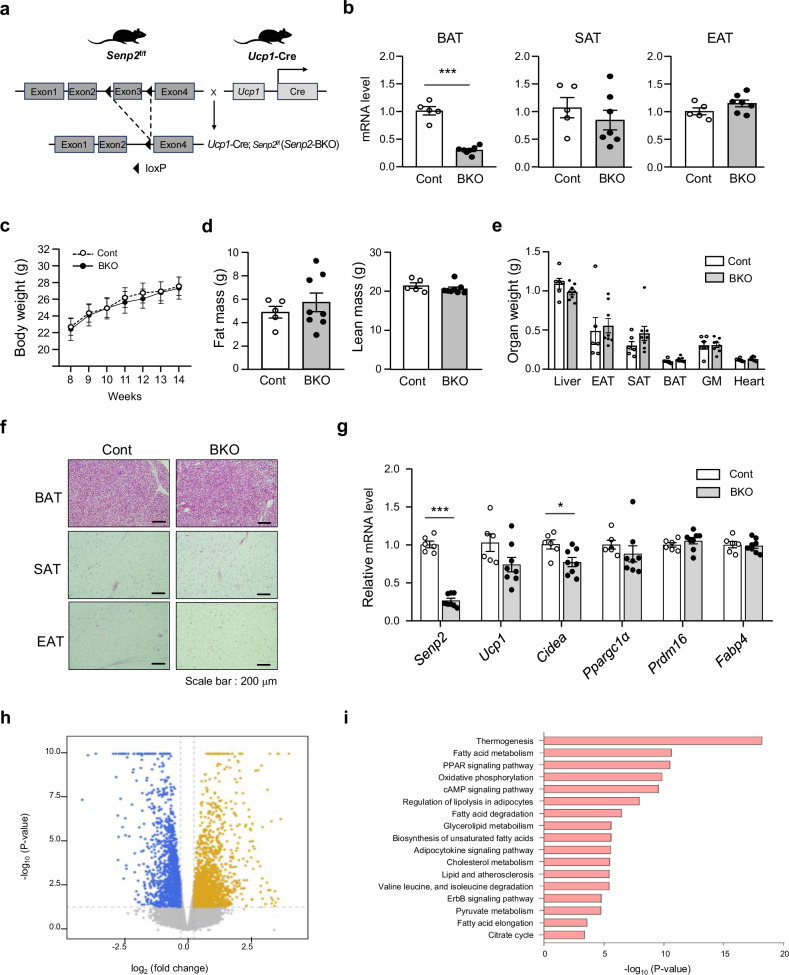
Brown fat-specific loss of SENP2 does not affect adipogenesis but affects expression of genes involved in thermogenesis and oxidative phosphorylation. **a** Generation of *Senp2*-BKO mice. **b** *Senp2* mRNA expression in adipose tissues of *Senp2*^f/f^ (Cont) and *Senp2*-BKO mice. **c**–**g** Control (Cont) and *Senp2*-BKO (BKO) mice were fed a standard chow diet for 16 weeks (*n* = 5–8): growth curves (**c**), fat mass and lean tissue weight (**d**), organ weight (**e**), H&E staining of adipose tissues (**f**) and gene expression profiles of BAT (**g**) in control and *Senp2*-BKO mice. All data are shown as the mean ± s.e.m. *P < 0.05 and *** *P* < 0.001 by Student’s *t*-test. **h**, **i** Volcano plot (**h**) and KEGG pathway (**i**) of the RNA-sequencing transcriptome in the BAT of control and *Senp2*-BKO mice (*n* = 3–4). Abbreviations: BAT brown adipose tissue, SAT subcutaneous adipose tissue, EAT epididymal adipose tissue, GM gastrocnemius muscle, Cont control.

To elucidate the role of SENP2 in BAT metabolism, we generated a brown adipocyte-specific *Senp2* knockout (*Senp2*-BKO) mouse model using *Ucp1*-Cre mice. In *Senp2*-BKO mice, exon 3 of the *Senp2* gene was excised using the Cre-LoxP recombination system (Fig. 1a). A marked reduction of *Senp2* mRNA expression in BAT of *Senp2*-BKO mice confirmed the deletion. *Senp2* mRNA expression was unaltered in other adipose regions, including subcutaneous adipose tissue (SAT) and epididymal adipose tissue (EAT) (Fig. 1b).

Both control and the *Senp2*-BKO mice fed a standard chow diet showed similar growth patterns (Fig. 1c). Measurements of body weight, body composition, and organ mass, including BAT, were comparable between the two groups at 12 weeks of age (Fig. 1c–e). Histological examination of BAT and WAT from control and *Senp2*-BKO mice showed no discernible differences (Fig. 1f). These data suggest that the BAT-specific SENP2 deletion did not affect the general growth or development of adipose tissues under normal dietary conditions. BAT marker gene expression analysis showed a slight decrease in expression of *Ucp1* and cell death-inducing DFFA-like effector A (*Cidea*) in BAT of *Senp2*-BKO mice. However, expression of PPARγ coactivator 1α (*Pgc1*α) and PR domain-containing 16 (*Prdm16*), important regulators of brown adipocyte differentiation, did not differ significantly between the groups (Fig. 1g). Consistently, there was no significant difference in the expression of representative BAT identity genes, including *Eva1*, *Lhx8*, *Zic1* and *Pat2* (Supplementary Fig. 1a).

SENP2 is predominantly located in the nucleus, where it regulates several transcription factors through deSUMOylation. RNA sequencing of BAT from control and *Senp2*-BKO mice revealed significant transcriptional changes. Among the 17,817 genes analyzed, 3,679 were differentially expressed: 1,845 genes were upregulated and 1,834 genes were downregulated in *Senp2*-BKO mice (Fig. 1h). KEGG enrichment analysis revealed that differentially expressed genes are associated with thermogenesis, fatty acid metabolism, PPAR signaling and oxidative phosphorylation pathways (Fig. 1i and Supplementary Fig. 1b). These findings indicated that SENP2 regulates metabolic and thermogenic processes in BAT.

In addition, the expression of UCP1 and several genes related to thermogenesis and glucose/lipid metabolism in SAT of *Senp2*-BKO mice were not different from those of control mice, suggesting that brown adipocyte-specific SENP2 knockout did not affect beige adipocyte differentiation and function (Supplementary Fig. 1c, d).

### Brown fat-specific SENP2 deficiency exacerbates insulin resistance in mice fed an HFD

**Fig. 2:**
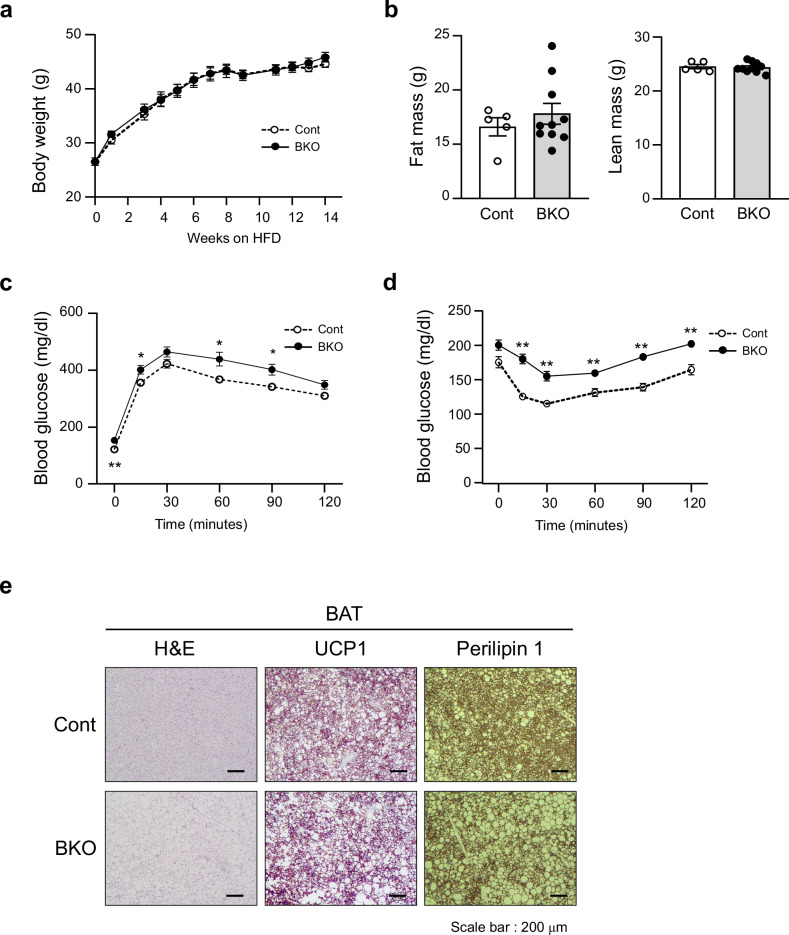
Brown fat-specific SENP2 deficiency exacerbates insulin resistance in mice fed an HFD. Control and *Senp2*-BKO mice were fed an HFD for 14 weeks (*n* = 5–10). **a**–**d** Body weights during 14 weeks of HFD feeding (**a**), fat mass and lean mass (**b**), GTT (**c**), and ITT (**d**) after 13 or 14 weeks of HFD feeding. **e** Representative H&E staining and immunohistochemistry of UCP1 and Perilipin 1 of BAT from control and *Senp2*-BKO mice. All data are shown as the mean ± s.e.m. *P < 0.05 and **P < 0.01 by Student’s *t*-test.

To investigate the role of SENP2 in BAT metabolism under energy overload conditions, we examined the metabolic outcomes of control and *Senp2*-BKO mice fed an HFD. There were no differences in body weight and fat percentages between the two groups of mice fed an HFD (Fig. 2a, b). However, *Senp2*-BKO mice exhibited increased systemic glucose intolerance after being on the HFD for 13 weeks, as determined by an intraperitoneal (i.p.) glucose tolerance test (Fig. 2c). The *Senp2*-BKO mice also exhibited exacerbated insulin resistance after being on the HFD for 14 weeks, as determined by the i.p. ITT (Fig. 2d). Histological analysis of BAT from *Senp2*-BKO mice fed an HFD showed an increase in enlarged lipid droplets, but no difference between control and *Senp2*-BKO mice was observed in SAT (Fig. 2e and Supplementary Fig. 2a). Although there was no obvious difference in the expression of some glucose- and lipid metabolism-related genes between control and BKO mice, expression of Neuregulin 4 (NRG4), a BAT-secreted factor known to regulate hepatic lipogenesis and insulin resistance, was reduced by SENP2 knockout (Supplementary Fig. 2b). These results indicate that SENP2 plays a role in maintaining the metabolic integrity of BAT and modulates systemic metabolism upon an HFD.

### Brown fat-specific loss of SENP2 impairs thermogenesis upon cold exposure

**Fig. 3:**
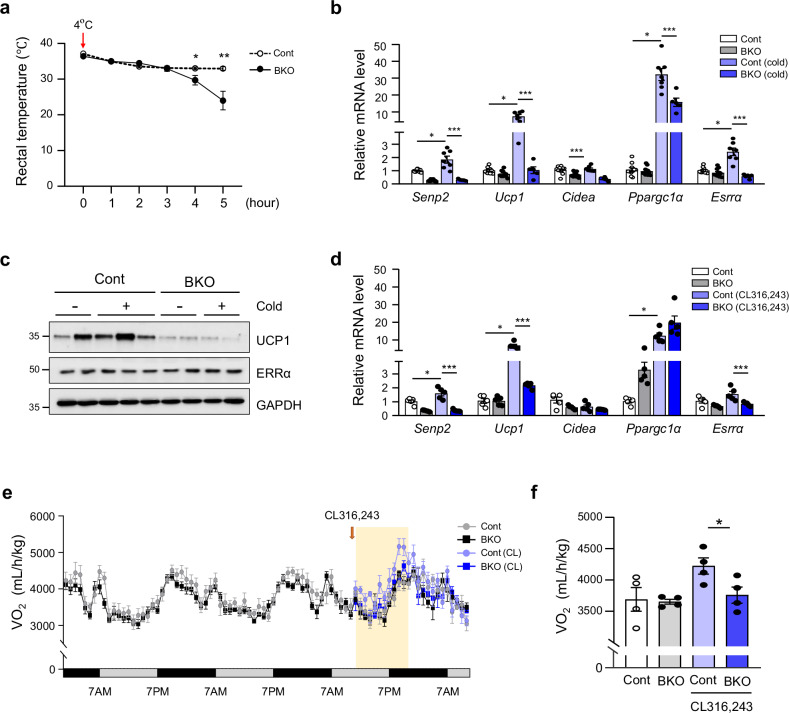
Brown fat-specific loss of SENP2 impairs thermogenesis upon cold exposure. **a** Core body temperature of control (Cont) and *Senp2*-BKO (BKO) mice during acute cold exposure at 4 °C for 5 h (*n* = 5–8). **b** Relative gene expression in BAT of control and *Senp2*-BKO mice after acute cold exposure determined by qPCR (*n* = 5–8). **c** Western blot analysis of UCP1 and ERRα of the BAT lysates. **d** Relative gene expression in BAT 6 h after injection of CL316,243 (10 mg/kg) (*n* = 5). The mRNA level of each gene in BAT from control mice without cold exposure or CL316,243 treatment was set to 1, and the others were expressed as its relative values. **e**, **f** Mice fed a standard chow diet were placed into individual cages in the Comprehensive Lab Animal Monitoring System for 2 days and then were injected with 10 mg/kg of CL316,243. Volume oxygen (VO_2_) consumption rates were measured (*n* = 4) (**e**) and the bar graphs represent the average VO_2_ across 12 h post-CL316,243 administration (the pale yellow region on **e**) (**f**). All data are shown as the mean ± s.e.m. Student’s *t*-test was applied (**a**) and an ANOVA followed by Tukey’s HSD test for multiple comparisons were performed during post hoc analyses (**b**–**f**). *P < 0.05, **P < 0.01 and ***P < 0.001.

As adaptive thermogenesis is the defining characteristic of BAT, we assessed the thermogenic capacity of control and *Senp2*-BKO mice in response to acute cold exposure. During exposure to 4 °C, both control and *Senp2*-BKO mice initially employed shivering to maintain body temperature. However, this initial response diminished after 2–3 h, and the body temperature of *Senp2*-BKO mice declined steeply, indicating impaired adaptive thermogenesis (Fig. 3a). Examination of gene expression in cold-activated BAT of control mice revealed upregulation of thermogenesis-associated genes, including *Ucp1* and *Pgc1α*, indicating activation of thermogenesis in response to cold exposure (Fig. 3b). By contrast, no significant increase in *Ucp1* expression upon cold exposure was observed in *Senp2*-BKO mice. This suggests that the absence of SENP2 impairs the ability of BAT to effectively upregulate the thermogenic pathways necessary for an adequate response to cold stress. UCP1 protein expression was significantly lower in BAT of *Senp2*-BKO mice compared with that of control mice, determined by western blot analysis (Fig. 3c). Changes in gene expression in BAT upon treatment with CL316,243, a β3-adrenoceptor agonist, were similar to those observed with acute cold exposure (Fig. 3d). In addition, i.p. injection of CL316,243 into control mice led to an increase in oxygen consumption indicating enhanced metabolic activity and thermogenesis (Fig. 3e, f). However, no increase in oxygen consumption was observed in *Senp2*-BKO mice following CL316,243 treatment. This absence of a metabolic response in *Senp2*-BKO mice confirms the impaired thermogenic capability of S*enp2*-BKO BAT and suggests that SENP2 plays an essential role in mediating complete activation of BAT.

### *Ucp1* transcription is regulated by SUMOylation of ERRα

**Fig. 4:**
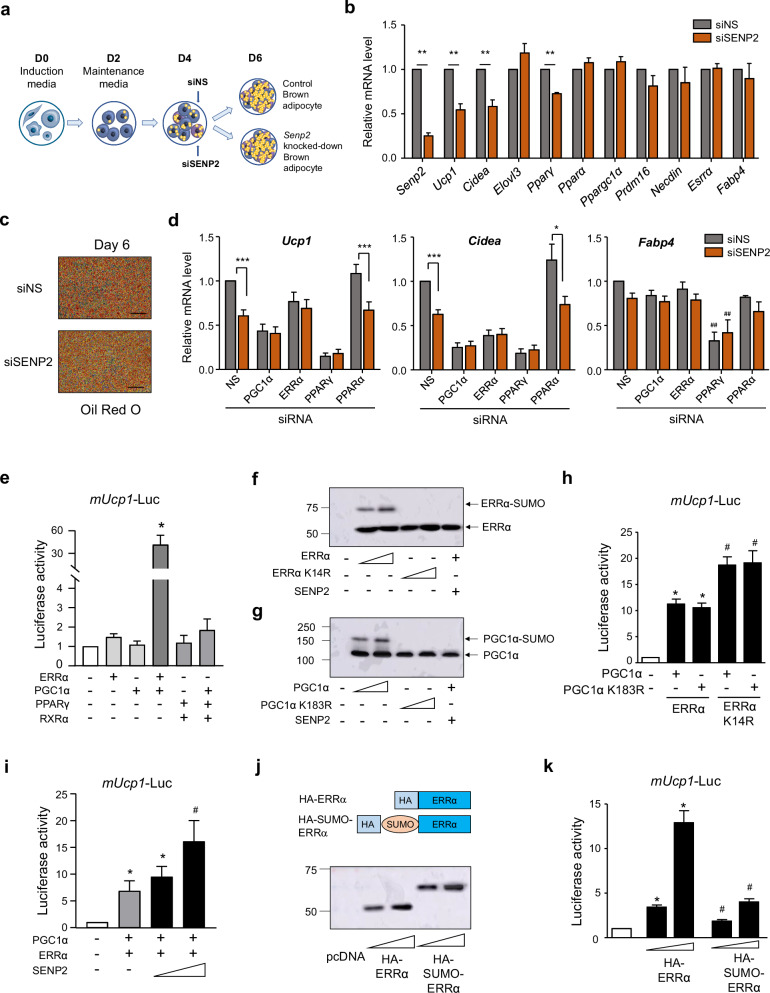
*Ucp1* transcription is regulated by SUMOylation of ERRα. **a** An experimental scheme of brown adipocyte differentiation and siRNA-induced knockdown of SENP2. **b** Relative mRNA levels of genes related to adipogenesis and thermogenesis in brown adipocytes upon SENP2 knockdown using siRNAs of SENP2 (siSENP2) (*n* = 5). **c**, Oil red O staining at day 6 of differentiation. **d** Relative mRNA levels of *Ucp1*, *Cidea* and *Fabp4* in response to knockdown of PGC1α, ERRα, PPARγ or PPARα. The values obtained from siNS-treated cells were set to 1, and the others were expressed as its relative values (*n* = 5). **e** Cos-7 cells were transfected with the *mouse Ucp1* promoter-Luc vector (m*Ucp1*-Luc) and expression vectors of ERRα, PGC1α, PPARγ or RXRα as indicated (*n* = 5). **f**, **g** Cos-7 cells were transfected with expression vectors of HA-ERRα, HA-ERRα K14R (**f**), or HA-PGC1α, HA-PGC1α K183R (**g**), together with SENP2 and SUMO1/UBC9 expression vectors. Western blotting was performed using an HA antibody. **h** Cos-7 cells were transfected with *mUcp1*-Luc and expression vectors as indicated (*n* = 3). **i**, Brown adipocyte cell line was used for transfection. The luciferase activity in the cells transfected with the reporter vector only was set to 1, and the others were expressed as its relative values. **j**, **k** After transfection of the indicated vectors, western blotting was performed with anti-HA antibody (**j**) and luciferase activities were measured (**k**) (*n* = 3). All data are expressed as the mean ± s.e.m. For multiple comparisons in **d**, data were analyzed using one-way ANOVA followed by Tukey’s HSD test for post hoc analysis. *P < 0.05, **P < 0.01, ***P < 0.001 and ^#^*P* < 0.05 versus siNS. *P < 0.05 versus the reporter only (**e**–**k**) and ^#^*P* < 0.05 versus ERRα (**h**–**k**).

As SENP2 knockout significantly affected thermogenesis and *Ucp1* expression upon cold exposure, we next investigated the mechanism by which SENP2 regulates *Ucp1* expression. Brown pre-adipocytes were differentiated and *Senp2* knockdown was achieved by transfecting cells with siRNA (siSENP2) on the fourth day of differentiation (Fig. 4a). qPCR analysis conducted on the sixth day showed downregulation of *Ucp1* and *Cidea* in the *Senp2*-knockdown brown adipocytes compared with control cells (Fig. 4b). Notably, knockdown of SENP2 did not affect *Fabp4* expression or formation of lipid droplets, indicating that SENP2 knockdown at this stage did not interfere with adipogenesis (Fig. 4b, c). Additionally, expression of genes related to mitochondrial biogenesis, fusion and fission remained unaffected by SENP2 knockdown and mitochondrial DNA copy numbers were similar in siSENP2-treated brown adipocytes and control cells (Supplementary Fig. 3a,b).

Further, we explored which transcription factors may be involved in reducing *Ucp1* mRNA expression. The siRNAs of PGC1α, ERRα, PPARγ and PPARα, which have been identified as SUMOylation targets, were co-transfected with siSENP2 (Fig. 4d and Supplementary Fig. 3c). Co-knockdown of SENP2 with PGC1α, ERRα or PPARγ, but not PPARα, abolished the effect of SENP2 knockdown on *Ucp1* and *Cidea* expression. Interestingly, the PPARγ knockdown reduced *Fabp4* mRNA expression, suggesting that the PPARγ knockdown impairs adipogenesis even on day 4 of differentiation. Considering that the SENP2 knockdown reduced mRNA expression of *Ucp1* and *Cidea* without inhibiting adipogenesis, these results suggest that SENP2 controls *Ucp1* transcription by modulating ERRα and PGC1α.

To explore how SENP2 regulates *Ucp1* transcription, Cos-7 cells were transiently transfected with the m*Ucp1*(−2,803/+213)-luciferase (Luc) reporter plasmid containing a 220 bp enhancer region at −2.5 kb of the *Ucp1* transcription start site. The enhancer region contains multiple *cis*-acting elements, including the PPAR response element (PPRE), the cAMP-response element (CRE) and the ERR response element (ERRE)^20–22^. Individual transfection of PPARγ/RXRα, ERRα and PGC1α expression vectors modestly induced *Ucp1* promoter activity, whereas transfection with both ERRα and PGC1α expression vectors significantly induced *Ucp1* promoter activity (Fig. 4e). We confirmed that the primary SUMOylation sites on ERRα and PGC1α are Lys 14 and Lys 183, respectively, and we previously showed that SENP2 deSUMOylated ERRα and PGC1α specifically^17^ (Fig. 4f, g). Transfection of the PGC1α K183R vector did not further enhance *Ucp1* promoter activity compared with transfection with wild-type PGC1α. However, co-transfection with the ERRα K14R and PGC1α (wild type or mutant) vectors increased *Ucp1* promoter activity compared with transfection with wild-type ERRα, suggesting that deSUMOylation of ERRα regulates *Ucp1* promoter activity (Fig. 4h). Consistent with these data, addition of SENP2 to wild-type ERRα and PGC1α further increased *Ucp1* promoter activity in the brown adipocyte cell line (Fig. 4i). To further examine the effect of ERRα SUMOylation, we constructed a SUMO–ERRα fusion vector (Fig. 4j), and we observed that *Ucp1* promoter activity increased with increased ERRα expression, but not with increased SUMO–ERRα expression (Fig. 4k). These results indicate that SENP2 enhances *Ucp1* promoter activity primarily through deSUMOylation of ERRα.

### SUMOylation of ERRα impairs cAMP-induced *Ucp1* transcription

**Fig. 5:**
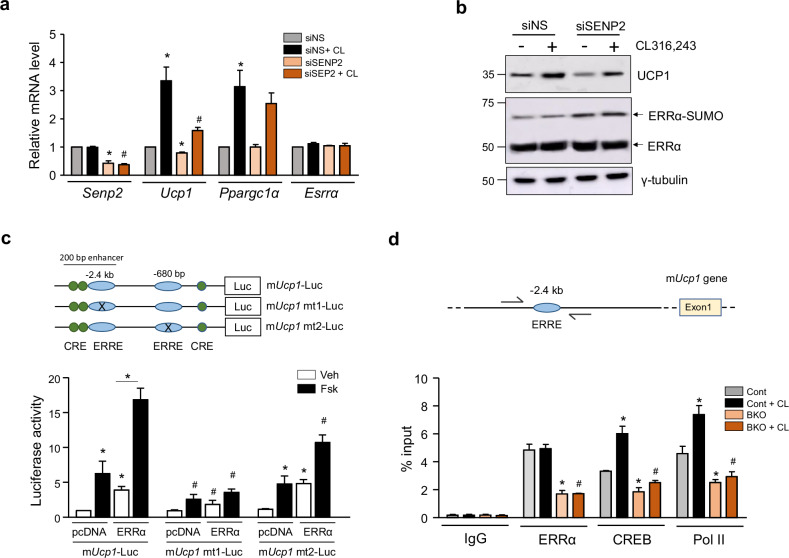
SUMOylation of ERRα impairs cAMP-induced *Ucp1* transcription. **a**, **b** Brown adipocytes transfected with siNS or siSENP2 were exposed to CL316,243 (CL) for 5 h before collection: expression of the indicated genes were determined by qPCR (*n* = 3) (**a**) and cell lysates were subjected to western blots (**b**). *P < 0.05 versus siNS, ^#^*P* < 0.05 versus siNS + CL. **c** Brown adipocytes were transfected with one of the m*Ucp1* promoter-Luc vectors and expression vectors for ERRα and PGC1α, and then treated with forskolin (Fsk) for 18 h (*n* = 3). *P < 0.05 versus pcDNA/Vehicle (Veh), ^#^*P* < 0.05 versus m*Ucp1*-Luc. **d** BAT of control (Cont) or *Senp2*-BKO (BKO) mice treated with CL316,243 (CL) were subjected to ChIP using antibodies against control IgG, ERRα, CREB and Pol II, followed by qPCR using the primers flanking the ERRE in the enhancer region. The data are shown as the mean ± s.e.m. of three independent experiments, *P < 0.05 versus Cont, ^#^*P* < 0.05 versus Cont *+* CL.

Next, we determined whether SUMOylation of ERRα affects *Ucp1* expression in response to acute cold stimuli. In concordance with the *Senp2*-BKO mice data shown in Fig. 3d, SENP2 knockdown attenuated the upregulation of *Ucp1* expression in brown adipocytes upon treatment of CL316,243 (Fig. 5a). In addition, upon SENP2 knockdown, ERRα SUMOylation increased, but ERRα levels remained stable (Fig. 5a, b).

Within the 2.8 kb 5′-flanking DNA of mouse *Ucp1 (mUcp1)*, we identified two regions containing sequences similar to the consensus ERRE (Fig. 5c, top). To determine the specific ERRα binding sites, brown adipocytes were transfected transiently with the wild-type *Ucp1* promoter-Luc plasmid or constructs lacking either the distal (−2.4 kb) or proximal (−680 bp) putative ERRE. Overexpression of ERRα, coupled with forskolin, an activator of the cAMP pathway, led to a 15-fold increase in wild-type *Ucp1* promoter activity. This induction was significantly diminished by the distal ERRE mutation, but not by the proximal ERRE mutation, indicating that ERRα binds to the distal ERRE in the 220 bp enhancer region 5′ of *Ucp1* (Fig. 5c). Next, we performed ChIP-qPCR analysis of the distal ERRE using BAT extracted from control and *Senp2*-BKO mice (Fig. 5d). Increased binding of ERRα to ERRE was observed in BAT from control mice compared with BAT from *Senp2*-BKO mice. Furthermore, administration of CL316,243 increased CREB and Pol II interactions with the ERRE complex in BAT from control mice; however, this enhanced binding was absent in BAT from *Senp2*-BKO mice. These results indicate that SENP2 deficiency impairs recruitment of the CREB and Pol II transcriptional activators to the *Ucp1* promoter following β3-adrenergic stimulation.

### SUMOylation of ERRα inhibits its DNA-binding ability

**Fig. 6:**
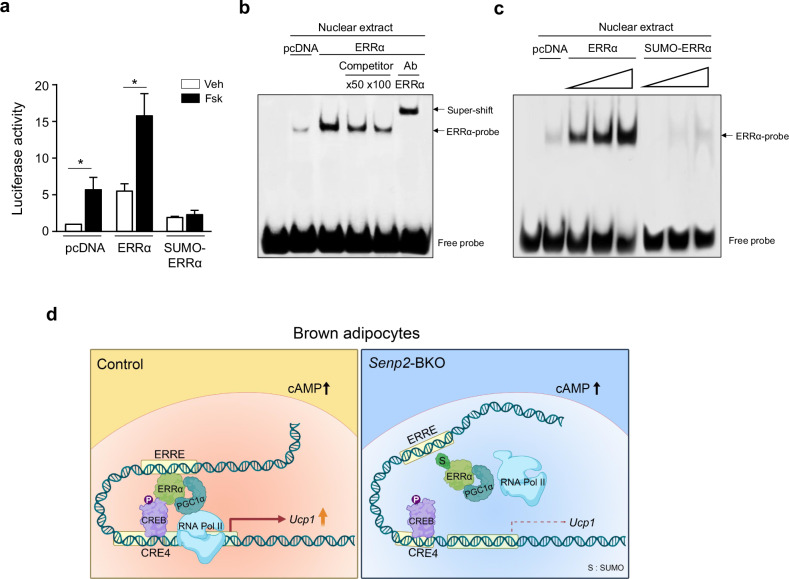
SUMOylation of ERRα inhibits its DNA-binding ability. **a** Brown adipocytes were transfected with the m*Ucp1*-Luc and expression vectors of ERRα or SUMO–ERRα in the presence of PGC1α followed by forskolin treatment. The data are shown as the mean ± s.e.m. of three independent experiments. **b**, **c** Nuclear extracts prepared from Cos-7 cells transfected with pcDNA, expression vector of ERRα (**b**) or SUMO–ERRα (**c**) were subjected to EMSA with the biotin-labeled *Ucp1* ERRE probe. Unlabeled oligonucleotides (50- or 100-fold excess) were used as competitors. ERRα antibody was used for super-shifting. The first lane contains the probe only. **d** A conceptual model showing the regulation of *Ucp1* transcription by SENP2 in a cAMP-stimulated condition.

Next, we investigated whether SUMOylation affects ERRα activity. Cos-7 cells were transfected with the m*Ucp1*-Luc reporter along with expression vectors containing wild-type ERRα or the SUMO–ERRα fusion and were subsequently treated with forskolin. Upon forskolin treatment, *Ucp1* promoter activity increased in brown adipocytes co-transfected with wild-type ERRα; however, *Ucp1* promoter activity did not increase in the cells co-transfected with the SUMO–ERRα fusion vector (Fig. 6a). As ERRα binding to the ERRE of the *Ucp1* promoter was reduced in BAT from *Senp2*-BKO mice, as shown in Fig. 5d, we tested whether SUMOylation of ERRα impairs its DNA-binding ability. EMSA was conducted using nuclear proteins extracted from Cos-7 cells expressing either ERRα or ERRα–SUMO fusion using ERRE as the probe. Specific binding of ERRα to ERRE was confirmed by addition of unlabeled competitor or ERRα antibody (Fig. 6b). We observed that binding of wild-type ERRα to ERRE increased in a dose-dependent manner; however, no binding was observed with SUMO–ERRα, even at high concentrations (Fig. 6c). These results suggest that SUMOylation of ERRα impedes its binding to ERRE.

To summarize, upon an increase of cAMP resulting from activation of the β3-adrenergic signaling pathway, phosphorylated CREB binds to CRE in the *Ucp1* promoter and interacts with other transcription factors, including ERRα bound to ERRE in the enhancer, which facilitates formation of a stable transcription complex that recruits RNA Pol II. As a result, *Ucp1* transcription increased robustly in the BAT from control mice. However, SUMOylated ERRα cannot bind to ERRE in the enhancer, thus interactions between the transcription activators at the distal enhancer with the proximal promoter regions of *Ucp1* are impeded. Consequently, the increase in SUMOylated ERRα in the BAT of *Senp2*-BKO mice restricted transcriptional upregulation of *Ucp1* triggered by activation of β3-adrenergic signaling (Fig. 6d). Our data suggest that binding of ERRα to ERRE in the enhancer region is essential for cAMP-induced transcription of *Ucp1* and that ERRα binding to the ERRE is primarily regulated by SENP2.

## Discussion

In this study, we explored the regulatory role of SENP2 on BAT function. Although control and *Senp2*-BKO mice fed a standard chow diet displayed similar phenotypes, cold tolerance and insulin sensitivity were impaired and aggravated in *Senp2*-BKO mice under conditions of acute cold exposure and an HFD, respectively. In this study, we focused on the thermogenic change and determined that SENP2 modulates *Ucp1* gene expression in mature brown adipocytes through deSUMOylation of ERRα, thereby influencing the thermogenic efficiency of BAT.

Previously, we demonstrated that SENP2 enhances CIDEA expression by modulating ERRα^17^. CIDEA is highly expressed in brown adipocytes and regulates thermogenesis and energy balance^23–25^. Both *Cidea* and *Ucp1* promoters contain binding sites for ERRα, suggesting a common transcriptional regulatory mechanism. However, although *Ucp1* expression is upregulated in response to cold exposure and adrenergic stimulation, *Cidea* expression is not^26–28^. Here, we showed that the interaction between ERRα and CREB at the *Ucp1* promoter leads to cold-induced upregulation of *Ucp1* transcription and analyzed the effects of SENP2 on *Ucp1* expression. Consequently, we found that deSUMOylation of ERRα by SENP2 affects basal and cold-induced expression of *Ucp1*.

Several reports suggested that, the 220 bp enhancer region approximately 2.5 kb upstream of the *Ucp1* transcriptional start site is important for cAMP-induced *Ucp1* expression in addition to CREB binding to the *Ucp1* promoter^29^. However, the specific element(s) within the 220 bp enhancer region responsible for cold-induced activation was not clearly defined. This enhancer region contains binding sites for several transcription factors, including CREB and PPAR. We determined that an ERRE within the enhancer is essential for robust activation of the *Ucp1* promoter in response to cold.

ERR family members are orphan receptors and their roles in BAT thermogenesis have been demonstrated^30,31^. ERRα and PGC1α synergistically activate *Ucp1* and Sirtuin 3 (*Sirt3*) transcription and induce mitochondrial biogenesis^32–35^. Whole-body ERRα knockout mice displayed only a mild decrease in BAT mitochondrial content and oxidative function and maintained normal UCP1 induction in response to cold^31^. ERRα is the predominant ERR isoform in brown adipocytes, which also express ERRβ and ERRγ. Deletion of ERRα along with deletion of ERRβ and ERRγ result in significant impairments in both transcriptional and metabolic responses to adrenergic stimulation^31^. As ERRα, ERRβ, and ERRγ contain SUMOylation sites, SENP2 knockout may inhibit the activity of all ERR isoforms, resulting in the pronounced phenotype observed in *Senp2*-BKO mice. Further studies are required to determine the functional effect of deSUMOylation of ERRβ and ERRγ by SENP2. In addition, we observed a twofold transcriptional increase in both SENP2 and ERRα following 5 h of cold exposure (Fig. 3b), which emphasizes their important in vivo roles in enhancing thermogenic capacity in response to cold exposure condition.

SENP2 is abundant in both WAT and BAT during adipogenesis^14,15^. In a previous report by Liang et al., brown adipocyte differentiation was suppressed via disinhibition of *Necdin* in a conditional BAT-specific SENP2 knockout mouse model, which was generated using myogenic factor 5 (*Myf5*)-Cre^16^. Similarly, in our previous experiments using *Adipoq*-Cre to knock out *Senp2* in both BAT and WAT, we observed a significant reduction in BAT size and histological analysis showed a lower density of multilocular clusters^14^. As brown adipocytes originate from *Myf5*-expressing progenitors^36^, *Senp2* was probably knocked out in a very early stage of development in the *Myf5*-Cre knockout model. In the AdipoChaser mouse model, which is based on the adiponectin promoter, generation of brown adipocytes in BAT begins as early as embryonic day 10 (E10) and finishes by E16^37^. However, UCP1 expression in BAT is induced by E19 in mice^38^, thus the targeted *Senp2* deletion in mature brown adipocytes was achieved using *Ucp1*-Cre. This study extended our understanding of the role of SENP2 beyond adipogenic differentiation and demonstrated that the SENP2 deletion in mature brown adipocytes substantially affects metabolic responses under stress conditions, such as cold or an HFD.

The previous study by Lee et al. using adipocyte-specific SENP2 knockout mice generated by *Adipoq*-Cre showed enlarged lipid droplets in BAT^14^. However, it was unclear whether this phenotype resulted from SENP2 deletion or was a secondary effect of WAT browning resulting from SENP2 knockout in WAT, as SENP2 was depleted from both WAT and BAT in that model. While the previous study examined the effects of adipocyte-specific SENP2 knockout across both WAT and BAT, this study specifically investigates the role of SENP2 in mature brown adipocytes using an *Ucp1*-Cre model. This approach enables us to directly evaluate the role of SENP2 in regulating thermogenic capacity and metabolic function in BAT, eliminating potential confounding effects from WAT. In addition, this study elucidates the molecular mechanism through which SENP2 modulates BAT thermogenesis: SENP2 deSUMOylates ERRα, a key transcription for *Ucp1* expression, particularly under conditions of adaptive thermogenesis.

Although we have demonstrated that SENP2 modulates *Ucp1* gene expression in mature brown adipocytes via deSUMOylation of ERRα—largely explaining the reduced metabolic capacity of *Senp2*-BKO mice—glucose disposal in BAT can be decoupled from its thermogenic function. This raises the question of how SENP2 depletion exacerbates insulin resistance without substantially altering energy balance. While lipid- and glucose metabolism-related gene expression remained largely unchanged, *Nrg4* expression was significantly reduced in the BAT of *Senp2*-BKO mice, consistent with findings in standard chow-fed mice. *Nrg4* encodes NRG4, a BAT-enriched secreted factor (BATokine) that improves insulin sensitivity by suppressing hepatic lipogenesis^39^. These findings suggest that dysregulated BATokine secretion may contribute to the systemic glucose-metabolic dysfunction observed in *Senp2*-BKO mice. Further studies are required to elucidate the metabolic roles of BATokines in the context of SENP2 depletion.

In conclusion, SENP2 plays an essential role in BAT thermogenesis by modulating ERRα. Given the considerable amount of metabolically active BAT present in adult humans, SENP2 in BAT may serve as a therapeutic target to treat metabolic disorders.

## Supplementary information

Supplementary Information

## Data Availability

Data are available from the corresponding author upon reasonable request.
