# Supplementary material for: SENP2 regulates UCP1-dependent thermogenesis in brown adipocytes via deSUMOylation of ERRα
Source: Exp Mol Med. 2025 Jun 27;57(6):1283–93. doi: 10.1038/s12276-025-01458-5 (PMC12229480; doi:10.1038/s12276-025-01458-5)
Supplement: Supplementary file 1 — Supplementary Information [file 12276_2025_1458_MOESM1_ESM.pptx]

## Slide 1
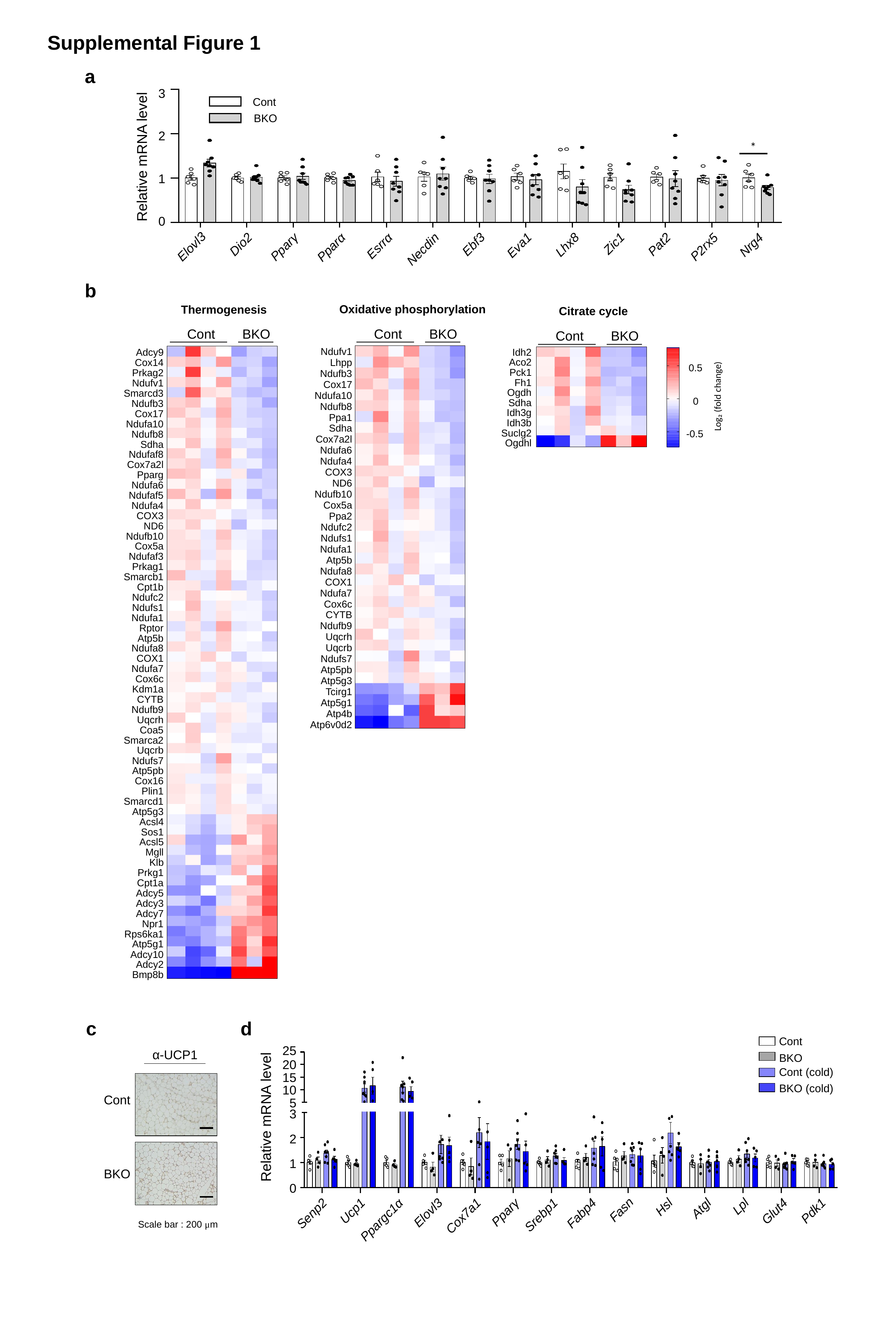

Supplemental Figure 1
a
3
Cont
BKO
2
*
Relative mRNA level
1
0
Zic1
Dio2
Ebf3
Pat2
Lhx8
Nrg4
Eva1
Esrrα
Elovl3
Pparγ
P2rx5
Pparα
Necdin
b
Oxidative phosphorylation
Thermogenesis
Citrate cycle
Cont
BKO
Cont
BKO
Cont
BKO
Adcy9
Cox14
Prkag2
Ndufv1
Smarcd3
Ndufb3
Cox17
Ndufa10
Ndufb8
Sdha
Ndufaf8
Cox7a2l
Pparg
Ndufa6
Ndufaf5
Ndufa4
COX3
ND6
Ndufb10
Cox5a
Ndufaf3
Prkag1
Smarcb1
Cpt1b
Ndufc2
Ndufs1
Ndufa1
Rptor
Atp5b
Ndufa8
COX1
Ndufa7
Cox6c
Kdm1a
CYTB
Ndufb9
Uqcrh
Coa5
Smarca2
Uqcrb
Ndufs7
Atp5pb
Cox16
Plin1
Smarcd1
Atp5g3
Acsl4
Sos1
Acsl5
Mgll
Klb
Prkg1
Cpt1a
Adcy5
Adcy3
Adcy7
Npr1
Rps6ka1
Atp5g1
Adcy10
Adcy2
Bmp8b
Ndufv1
Lhpp
Ndufb3
Cox17
Ndufa10
Ndufb8
Ppa1
Sdha
Cox7a2l
Ndufa6
Ndufa4
COX3
ND6
Ndufb10
Cox5a
Ppa2
Ndufc2
Ndufs1
Ndufa1
Atp5b
Ndufa8
COX1
Ndufa7
Cox6c
CYTB
Ndufb9
Uqcrh
Uqcrb
Ndufs7
Atp5pb
Atp5g3
Tcirg1
Atp5g1
Atp4b
Atp6v0d2
Idh2
Aco2
Pck1
Fh1
Ogdh
Sdha
Idh3g
Idh3b
Suclg2
Ogdhl
0.5
0
-0.5
Log₂ (fold change)
c
d
Cont
BKO
Cont (cold)
BKO (cold)
25
α-UCP1
20
15
10
Cont
5
Relative mRNA level
3
2
1
BKO
0
Lpl
Hsl
Atgl
Fasn
Pdk1
Ucp1
Glut4
Pparγ
Elovl3
Fabp4
Senp2
Srebp1
Cox7a1
Ppargc1α
Scale bar : 200 μm

## Slide 2
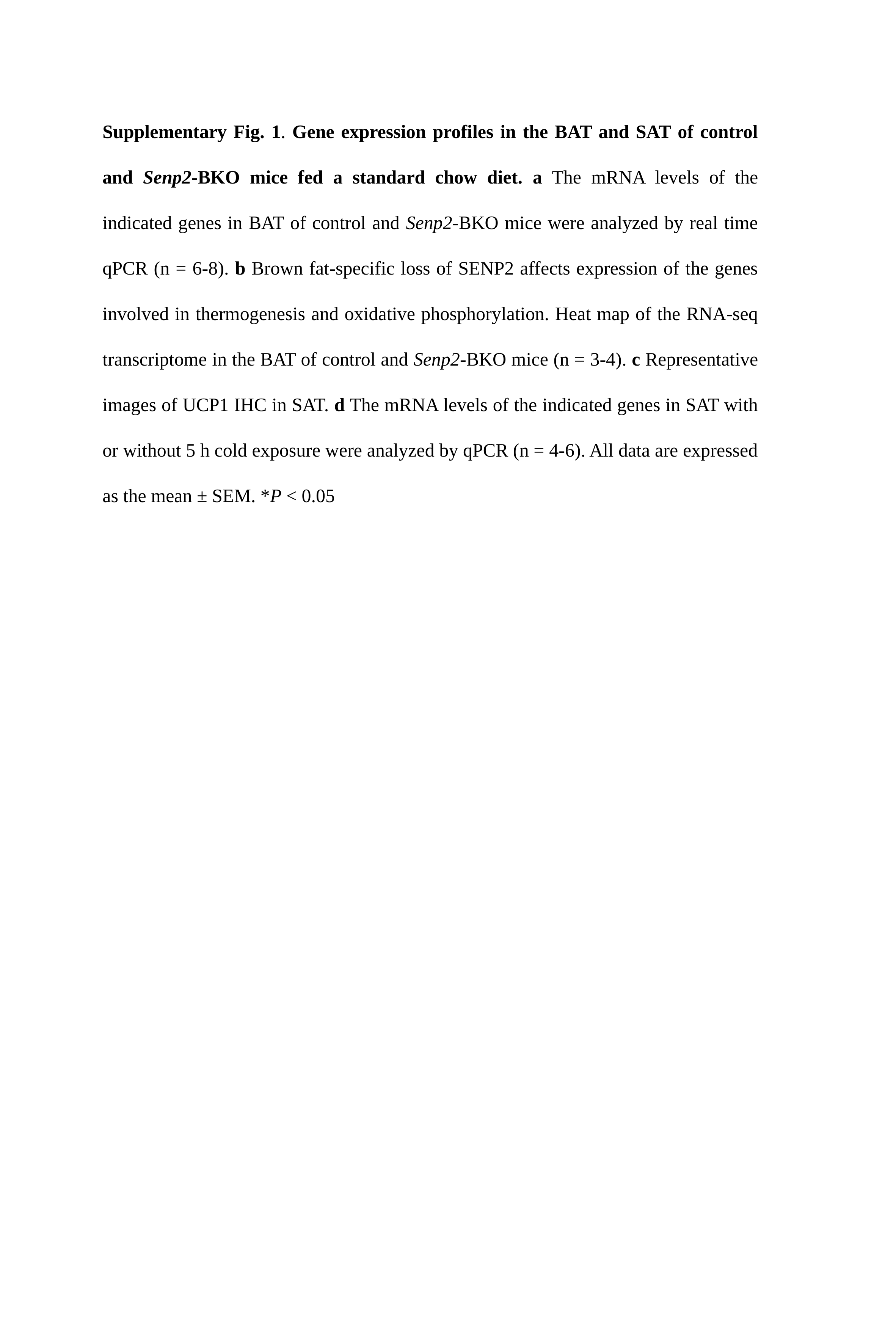

Supplementary Fig. 1. Gene expression profiles in the BAT and SAT of control and Senp2-BKO mice fed a standard chow diet. a The mRNA levels of the indicated genes in BAT of control and Senp2-BKO mice were analyzed by real time qPCR (n = 6-8). b Brown fat-specific loss of SENP2 affects expression of the genes involved in thermogenesis and oxidative phosphorylation. Heat map of the RNA-seq transcriptome in the BAT of control and Senp2-BKO mice (n = 3-4). c Representative images of UCP1 IHC in SAT. d The mRNA levels of the indicated genes in SAT with or without 5 h cold exposure were analyzed by qPCR (n = 4-6). All data are expressed as the mean ± SEM. *P < 0.05

## Slide 3
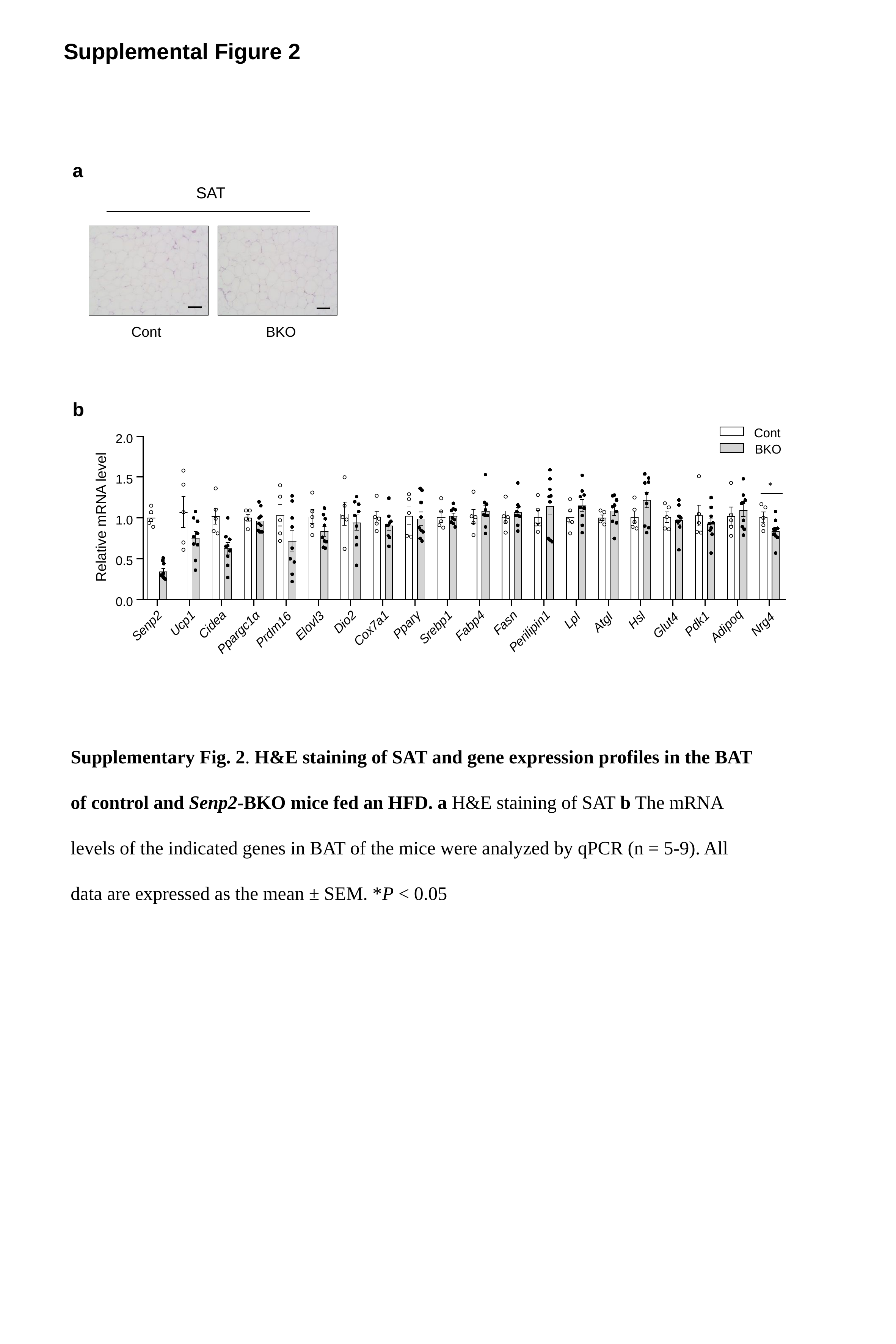

Supplemental Figure 2
a
SAT
Cont
BKO
b
Cont
BKO
2.0
1.5
*
Relative mRNA level
1.0
0.5
0.0
Hsl
Lpl
Dio2
Atgl
Fasn
Ucp1
Pdk1
Nrg4
Cidea
Pparγ
Glut4
Fabp4
Senp2
Elovl3
Adipoq
Srebp1
Cox7a1
Prdm16
Perilipin1
Ppargc1α
Supplementary Fig. 2. H&E staining of SAT and gene expression profiles in the BAT of control and Senp2-BKO mice fed an HFD. a H&E staining of SAT b The mRNA levels of the indicated genes in BAT of the mice were analyzed by qPCR (n = 5-9). All data are expressed as the mean ± SEM. *P < 0.05

## Slide 4
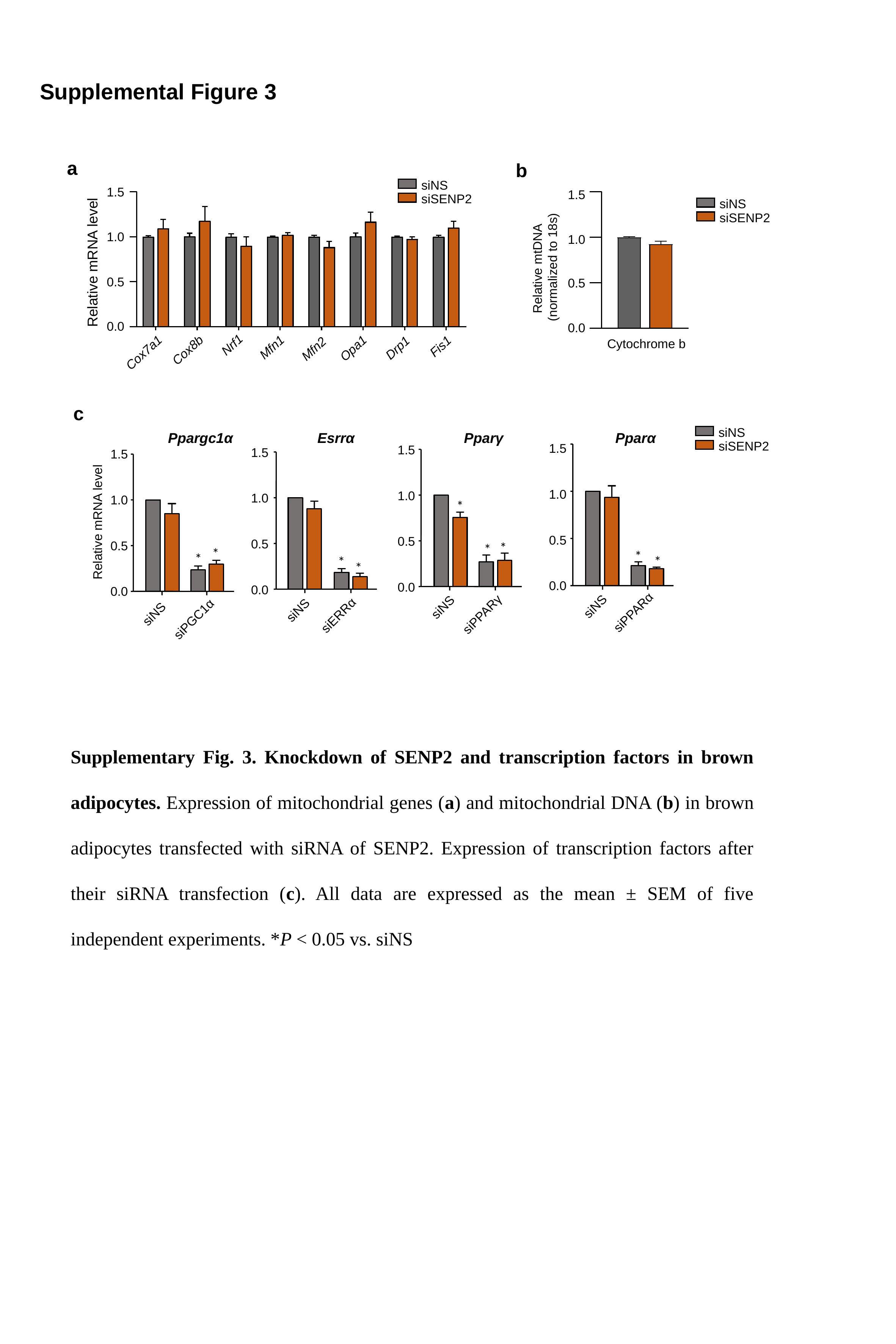

Supplemental Figure 3
a
b
siNS
siSENP2
1.5
1.5
siNS
siSENP2
1.0
1.0
Relative mtDNA
(normalized to 18s)
Relative mRNA level
0.5
0.5
0.0
0.0
Cytochrome b
Nrf1
Fis1
Mfn1
Drp1
Opa1
Mfn2
Cox8b
Cox7a1
c
siNS
siSENP2
Ppargc1α
Esrrα
Pparγ
Pparα
1.5
1.5
1.5
1.5
1.0
1.0
1.0
1.0
*
Relative mRNA level
0.5
0.5
*
0.5
*
0.5
*
*
*
*
*
*
0.0
0.0
0.0
0.0
siNS
siNS
siNS
siPPARα
siNS
siPPARγ
siERRα
siPGC1α
Supplementary Fig. 3. Knockdown of SENP2 and transcription factors in brown adipocytes. Expression of mitochondrial genes (a) and mitochondrial DNA (b) in brown adipocytes transfected with siRNA of SENP2. Expression of transcription factors after their siRNA transfection (c). All data are expressed as the mean ± SEM of five independent experiments. *P < 0.05 vs. siNS

## Slide 5
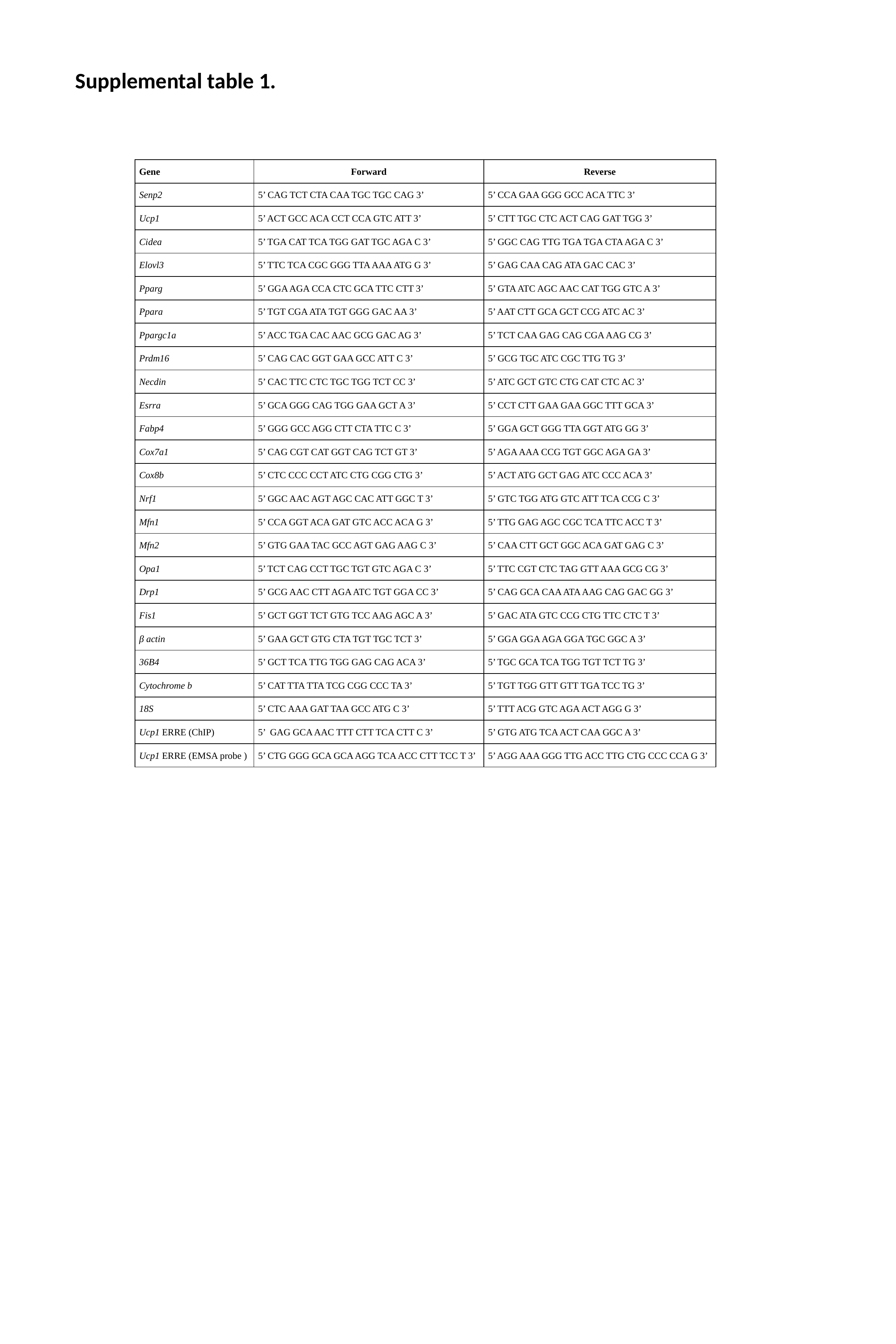

Supplemental table 1.
| Gene | Forward | Reverse |
| --- | --- | --- |
| Senp2 | 5’ CAG TCT CTA CAA TGC TGC CAG 3’ | 5’ CCA GAA GGG GCC ACA TTC 3’ |
| Ucp1 | 5’ ACT GCC ACA CCT CCA GTC ATT 3’ | 5’ CTT TGC CTC ACT CAG GAT TGG 3’ |
| Cidea | 5’ TGA CAT TCA TGG GAT TGC AGA C 3’ | 5’ GGC CAG TTG TGA TGA CTA AGA C 3’ |
| Elovl3 | 5’ TTC TCA CGC GGG TTA AAA ATG G 3’ | 5’ GAG CAA CAG ATA GAC CAC 3’ |
| Pparg | 5’ GGA AGA CCA CTC GCA TTC CTT 3’ | 5’ GTA ATC AGC AAC CAT TGG GTC A 3’ |
| Ppara | 5’ TGT CGA ATA TGT GGG GAC AA 3’ | 5’ AAT CTT GCA GCT CCG ATC AC 3’ |
| Ppargc1a | 5’ ACC TGA CAC AAC GCG GAC AG 3’ | 5’ TCT CAA GAG CAG CGA AAG CG 3’ |
| Prdm16 | 5’ CAG CAC GGT GAA GCC ATT C 3’ | 5’ GCG TGC ATC CGC TTG TG 3’ |
| Necdin | 5’ CAC TTC CTC TGC TGG TCT CC 3’ | 5’ ATC GCT GTC CTG CAT CTC AC 3’ |
| Esrra | 5’ GCA GGG CAG TGG GAA GCT A 3’ | 5’ CCT CTT GAA GAA GGC TTT GCA 3’ |
| Fabp4 | 5’ GGG GCC AGG CTT CTA TTC C 3’ | 5’ GGA GCT GGG TTA GGT ATG GG 3’ |
| Cox7a1 | 5’ CAG CGT CAT GGT CAG TCT GT 3’ | 5’ AGA AAA CCG TGT GGC AGA GA 3’ |
| Cox8b | 5’ CTC CCC CCT ATC CTG CGG CTG 3’ | 5’ ACT ATG GCT GAG ATC CCC ACA 3’ |
| Nrf1 | 5’ GGC AAC AGT AGC CAC ATT GGC T 3’ | 5’ GTC TGG ATG GTC ATT TCA CCG C 3’ |
| Mfn1 | 5’ CCA GGT ACA GAT GTC ACC ACA G 3’ | 5’ TTG GAG AGC CGC TCA TTC ACC T 3’ |
| Mfn2 | 5’ GTG GAA TAC GCC AGT GAG AAG C 3’ | 5’ CAA CTT GCT GGC ACA GAT GAG C 3’ |
| Opa1 | 5’ TCT CAG CCT TGC TGT GTC AGA C 3’ | 5’ TTC CGT CTC TAG GTT AAA GCG CG 3’ |
| Drp1 | 5’ GCG AAC CTT AGA ATC TGT GGA CC 3’ | 5’ CAG GCA CAA ATA AAG CAG GAC GG 3’ |
| Fis1 | 5’ GCT GGT TCT GTG TCC AAG AGC A 3’ | 5’ GAC ATA GTC CCG CTG TTC CTC T 3’ |
| β actin | 5’ GAA GCT GTG CTA TGT TGC TCT 3’ | 5’ GGA GGA AGA GGA TGC GGC A 3’ |
| 36B4 | 5’ GCT TCA TTG TGG GAG CAG ACA 3’ | 5’ TGC GCA TCA TGG TGT TCT TG 3’ |
| Cytochrome b | 5’ CAT TTA TTA TCG CGG CCC TA 3’ | 5’ TGT TGG GTT GTT TGA TCC TG 3’ |
| 18S | 5’ CTC AAA GAT TAA GCC ATG C 3’ | 5’ TTT ACG GTC AGA ACT AGG G 3’ |
| Ucp1 ERRE (ChIP) | 5’ GAG GCA AAC TTT CTT TCA CTT C 3’ | 5’ GTG ATG TCA ACT CAA GGC A 3’ |
| Ucp1 ERRE (EMSA probe ) | 5’ CTG GGG GCA GCA AGG TCA ACC CTT TCC T 3’ | 5’ AGG AAA GGG TTG ACC TTG CTG CCC CCA G 3’ |
